# DNAp: A Pipeline for DNA-seq Data Analysis

**DOI:** 10.1038/s41598-018-25022-6

**Published:** 2018-05-01

**Authors:** Jason L. Causey, Cody Ashby, Karl Walker, Zhiping Paul Wang, Mary Yang, Yuanfang Guan, Jason H. Moore, Xiuzhen Huang

**Affiliations:** 1 Department of Computer Science, Arkansas State University, Jonesboro, Arkansas 72467 United States of America; 2 Department of Biomedical Informatics and the Myeloma Institute, University of Arkansas for Medical Sciences, Little Rock, Arkansas 72205 United States of America; 3 Department of Mathematics and Computer Science, University of Arkansas at Pine Bluff, Pine Bluff, Arkansas 55455 United States of America; 4 Institute for Biomedical Informatics, University of Pennsylvania, Philadelphia, Pennsylvania 19104 United States of America; 5 Department of Information Science, University of Arkansas at Little Rock, Little Rock, Arkansas 72204 United States of America; 6 Department of Computational Medicine & Bioinformatics, University of Michigan, Ann Arbor, Michigan 48109 United States of America

## Abstract

Next-generation sequencing is empowering genetic disease research. However, it also brings significant challenges for efficient and effective sequencing data analysis. We built a pipeline, called DNAp, for analyzing whole exome sequencing (WES) and whole genome sequencing (WGS) data, to detect mutations from disease samples. The pipeline is containerized, convenient to use and can run under any system, since it is a fully automatic process in Docker container form. It is also open, and can be easily customized with user intervention points, such as for updating reference files and different software or versions. The pipeline has been tested with both human and mouse sequencing datasets, and it has generated mutations results, comparable to published results from these datasets, and reproducible across heterogeneous hardware platforms. The pipeline DNAp, funded by the US Food and Drug Administration (FDA), was developed for analyzing DNA sequencing data of FDA. Here we make DNAp an open source, with the software and documentation available to the public at http://bioinformatics.astate.edu/dna-pipeline/.

## Introduction

Next generation sequencing (NGS) is becoming the most popular high throughput technology for biological and biomedical research, including the study of various genetic diseases as well as drug design and discovery for the diseases. DNA-seq data analysis is to study genomic variants through aligning raw reads from NGS sequencing to a reference genome and then apply variant call software to identify genomic mutations, including SNPs, etc. However, the analysis of the sequencing data brings significant challenges to the community^1–3^: (i) the analysis requires in-depth knowledge and understanding of the needed bioinformatics software packages; (ii) it needs access to High-performance computing resources; (iii) the analysis needs to be consistent and be able to reproduce the results to facilitate comparisons as well as downstream analysis.

Especially in a large research collaboration, the DNA-seq analysis introduces additional challenges. It is ideal that all collaborators are using the same versions of each software package in the pipeline, and with the same tuning parameters. This could be achieved by carefully defining software versions and parameters and leaving each collaborator responsible for ensuring that the correct versions are installed and that the correct parameters are used when executing each tool in the pipeline; but this requires a high level of technical expertise and attention to detail at every collaboration site. It is also possible to containerize each individual tool. This ensures correct versioning of tools, but can still allow for mistakes in tuning parameters or connections between pipeline stages, depending on implementation. Our approach was to create an end-to-end pipeline environment in a container to allow every collaborator to operate in the same virtual environment with the same software (down to the operating system level), and provide predetermined analysis sequences so that the set of tools used and all tuning parameters can be hard-coded and shared between all collaborators, removing the need for end-users running analyses to follow complicated protocols and risk entering incorrect parameters. After the pipeline is configured, every analysis run follows identical, pre-determined steps. The operator need only specify the type of analysis to perform.

Currently there are many publications regarding the WGS/WES data analysis pipelines, and some publications are focused on software or method comparisons for the pipelines^1,2^. Most of the published pipelines are not end-to-end pipelines, i.e., they do not cover the entire data analysis process^3^. We developed the DNAp pipeline to demonstrate the feasibility of an end-to-end containerized tool for WGS/WES analysis and tested with human and mouse sequencing data and compared with published results, and by repeating an analysis on the same human patient on heterogeneous platforms. We have prepared a Docker image bundle containing the pipeline and made it available to the public at: http://bioinformatics.astate.edu/dna-pipeline/.

Several tools exist for building genomic analysis pipelines, including Galaxy^4–6^, Seven Bridges (https://www.sevenbridges.com/platform/), ExScalibur^3^, and bcbio-nextgen^7^. Galaxy is an open-source platform designed to be run in a central location, with access via a web-based interface; installing in separate sites is possible but would re-introduce software versioning differences. Galaxy supports transparent pipelines and reproducible analyses^6^. The user-friendly interface, large set of available tools, and wealth of online support make Galaxy a compelling choice for many applications. Seven Bridges is a proprietary platform with a web-based interface. It includes many configurable tools and offers reproducibility via detailed logging of the analyses that can be re-played (source: https://www.sevenbridges.com/platform/). Researchers must pay an annual fee plus compute and storage costs (passed through from AWS or Google Cloud) to make use of the Seven Bridges platform, and it is not self-hostable. ExScalibur, specifically ExScaliburSMD (https://github.com/cribioinfo/ExScaliburSMD)), supporting somatic variant calling with a pipeline designed to run on Amazon AWS. Of these options, it is most similar to our pipeline, however since our pipeline is packaged as a Docker container it can be executed in self-hosted settings as well as cloud-hosted settings without requiring multiple installations of each individual tool. Bcbio-nextgen is a Python toolkit that provides multiple best-practice pipelines; it requires installation on a workstation or server. While bcbio-nextgen itself isn’t contained in a Docker instance be default, it includes a tool to create and deploy Docker or AWS cloud instances of analyses runs.

## Methods

### Overview of the DNAp Pipeline

The DNAp Pipeline is a Docker (http://www.docker.com) container image based on the Centos 7 operating system, with tools for end-to-end Whole Genome and Whole Exome analysis. The software tools were selected according to recommendations from the Broad Institute Best Practices guide^8^, with customizations as requested by our collaborators in the project for which the pipeline was developed. Pipeline analysis orchestration (chaining of stages, management of inputs and outputs, logging, etc.) is managed by the Bpipe software package. A listing of software packages included is shown in Table 1.

**Table 1:** The full list of software tools available in the DNAp pipeline is shown in relative order of execution in the first column.

Tool	WES Mode	WGS Mode
FastQC	*	*
BWA-MEM	*	*
Picard Tools SortSam	*	*
Picard Tools BuildBamIndex	*	*
Picard Tools MarkDuplicates	*	*
Picard Tools MergeSamFiles	*	*
GATK RealignerTargetCreator	optional	optional
GATK IndelRealigner	optional	optional
GATK BaseRecalibrator	optional	optional
GATK AnalyzeCovariates	optional	optional
GATK PrintReads	optional	optional
GATK DiagnoseTargets	*	
GATK DepthOfCoverage		*
Qualimap	*	*
Strelka	*	*
MuTect2	*	*
GATK CatVariants	*	*
Samtools view		*
Samtools sort		*
Lumpy		*
bcftools filter		*
Picard Tools SortVcf		*
Breakdancer		*
Pindel		*
pindel2vcf		*
Jaquard merge	*	*
GATK SelectVariants	*	*
GATK CombineVariants		*
SnpEff	*	*
Oncotator	*	*

The second and third columns indicate which tools are utilized by default in the Whole Exome and Whole Genome pipeline analysis modes, respectively. Some tools are used multiple times, but for brevity each is listed only at the relative point of first use. Tools listed as “optional” are executed only if realignment around indels is requested.

The pipeline container is designed to be run as an interactive “virtual machine”, allowing researchers to quickly set up identical analysis environments on a diverse range of host machines. Once logged into the container environment, customizable commands are provided for running various analyses. All installed tools are also available for individual use as they would be on a stand-alone machine. By making specified host directories visible to the running container, input files are immediately available in the container, and outputs are automatically placed on the host’s storage by the pipeline. Analysis settings and custom configurations can be applied by placing configuration files into predefined locations in the host’s shared directory structure before launching the container.

The pipeline container supports several pre-configured analysis sequences to be run using built-in “helper” software run-pipeline. Supported sequences are: Run QC on FastQ files, trim adapters from reads, QC and alignment of reads (with or without realignment around known indels), realignment around indels given an input BAM file, processing a BAM file to call and annotate variants (with or without realignment), annotation of variant calls with SnpEff^9^, annotation of variant calls with Oncotator^10^, and full end-to-end processing from FastQ inputs through variant calling and annotation (with or without realignment). Additionally, the full suite of GATK^11^ software, Picard Tools^12^, SAMtools^13^, BCFtools^13^,, Jaquard^14^, BWA-MEM^15^, Strelka^16^, Mutect2^17^, Qualimap^18^, FastQC^19^, SnpEff^9^, and Oncotator^10^ are installed and available as stand-alone command line tools.

### Benefits of the end-to-end container approach

The end-to-end container represents a complete virtual analysis environment that is identical at every site. All software from the operating system to the individual analysis tools is the same; all sites share the same image and use it unmodified throughout the research project, ensuring uniform results^20^. Setup is easy, since the only software that needs to be installed on the host system is Docker (and the optional helper “startup” script); the container image is then imported and is immediately in a ready-to-run state. Because it is a single container, less technical expertise is required to make use of the system; in fact, a “startup” script can be supplied to allow the user to run a single simple command to enter the environment (starting the Docker container and attaching to the correct data volumes). Once inside the environment, complete analyses tasks are automated by another set of simple commands (running individual tools is also possible if needed). Finally, portability is a major advantage. The Docker platform is now supported on Linux, Windows, and Mac OS platforms as well as the major “cloud” platforms (Amazon Web Services, Google Cloud Platform, Microsoft Azure). The end-to-end container can be deployed virtually anywhere without worrying about compatibility of individual tool versions.

### Limitations of the end-to-end container approach

Using a single container for the entire pipeline does introduce challenges for tool customization and parameter tuning. To change a tool, the new tool must be installed in the running container, then the Bpipe configuration (used to automate the pipeline stages) must be updated and the resulting modifications to the container must be “committed” to create a new version of the container image. This requires some additional knowledge of the Docker development process, but it only needs to be done once — the resulting container image is then distributed to all collaboration sites in a ready-to-use state. Another possible limitation of the container option versus installing tools directly on the host system is that end users must learn to launch the container and enter the virtual environment before starting analyses; we try to overcome this challenge by supplying a “startup” script that performs the complex Docker container commands automatically given a simpler command from the user. Additionally, some tools parallelize differently than others. When running on i.e. a computing cluster, being able to request different resources from the queuing system for different pipeline stages would be an advantage. The monolithic container must be scheduled with a maximum allocation of resources (memory, processors, etc.) for the duration of the analysis, even though some stages cannot utilize the allocation well. One mitigation could be to create “staged” analysis runs so that the pipeline would perform only a certain subset of the full analysis in each stage; stages could then be scheduled according to the resource requirements of that stage. Finally, even though the container ensures a common software environment, some tools within the pipeline may still perform differently depending on the hardware environment (i.e. number of CPUs, amount of memory, etc.).

### Equipment

The DNAp Pipeline can be executed on any computer hardware that is capable of running Docker. Memory and storage requirements are the main hardware limitation for a DNA pipeline. We recommend a minimum of 20 GB of storage for the host operating system running Docker, and a minimum of 1.5 TB of secondary storage for the data inputs and outputs in the working directory (likely more if you are doing a WGS analysis with high coverage). The pipeline will automatically take advantage of multiple CPUs if they are available; we recommend a minimum of 8 processing cores.

### Software setup

Docker must be installed on the host machine. The installation of Docker is described on the Docker website (https://www.docker.com) and is beyond the scope of this protocol.

Import the DNAp pipeline image bundle (available at http://bioinformatics.astate.edu/dna-pipeline/) into Docker. The downloaded archive contains setup instructions and a “helper” script pipeline-start.sh that will be used to launch the container.

We have included additional details about configuration of the directory structure and locations for input and reference data files in the Supplementary Material.

### Running a Pre-Configured Pipeline

Running a preset sequence of pipeline stages is accomplished with the run-pipeline command within the running container. The command will start Bpipe^21^ with the correct entry point and arguments automatically, given WES or WGS mode and (for some commands) files to operate on. Most commands will use defaults for the file lists. The command run-pipeline -h can be used to show available options. More information is available in the Supplemental Material.

### Procedure: Human WES

**Figure 1:**
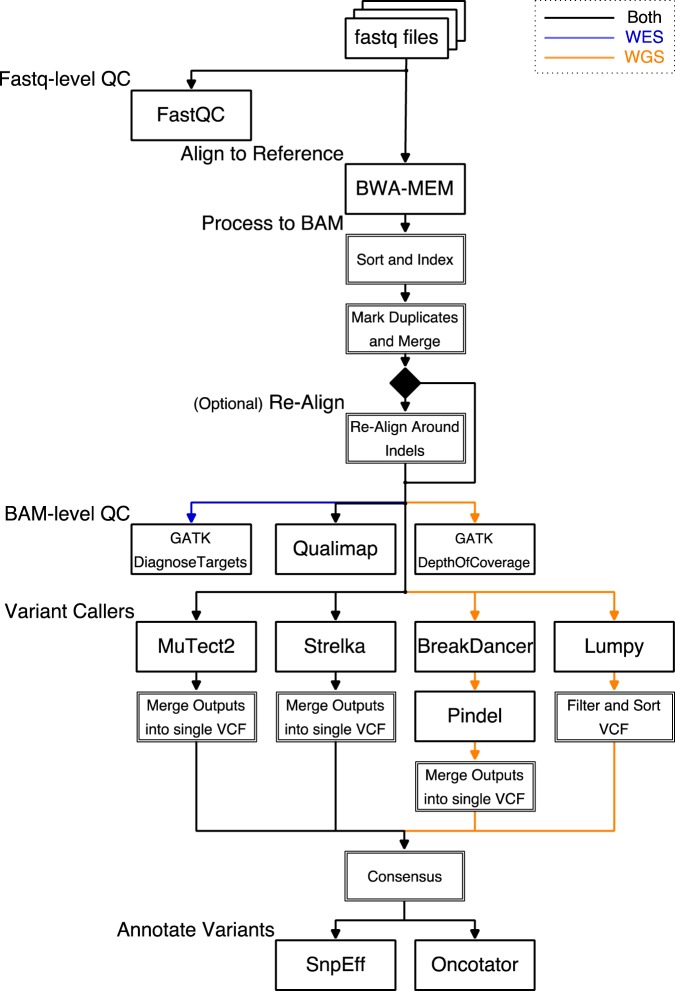
The DNAp pipeline processes from fastq-format input files to annotated VCF outputs, either in Whole Exome or Whole Genome analysis modes, with options for runnings specific parts of the pipeline as needed. The diagram above shows end-to-end operation; major tools are named and intermediate processing is shown as composite processes (double lines). Data flow paths that are specific to the WES or WGS modes are color coded blue for WES-only and orange for WGS-only.

This procedure runs the full pre-configured Whole Exome pipeline from *fastq* inputs to produce annotated variant call outputs for a Whole Exome analysis. The overall flow diagram is shown in Fig. 1.

1. Place inputs (*fastq* reads) into the input directory (fastq as shown above).
2. Create a directory named wes_bed inside the “working” directory (working_dir as shown above).
3. Place the BED file describing the intervals included in your Whole Exome enrichment kit into the wes_bed directory and name the file regions.bed.
4. Launch the pipeline container with./pipeline-start.sh.
5. Verify that the *fastq* files are in place in the /Results/fastq/ directory and that the file /Results/wes_bed/regions.bed exists in the running container.
6. Start the full-run pipeline with run-pipeline WES full.
7. Monitor the output from Bpipe (see Bpipe^21^ documentation for more information).
8. When the pipeline exits, you may exit the container with the exit command. All outputs will be located in the working_dir directory on the host machine.

This procedure runs the full end-to-end WES analysis; you can also run incremental analyses using the run-pipeline command. Enter run-pipeline -h for information about other pre-configured options.

### Procedure: Human WGS

This procedure runs the full pre-configured Whole Genome pipeline from *fastq* inputs to produce annotated variant call outputs. The overall flow diagram is shown in Fig. 1.

1. Place inputs (*fastq* reads) into the input directory (fastq as shown above).
2. Launch the pipeline container with./pipeline-start.sh.
3. Verify that the *fastq* files are in place in the /Results/fastq/ directory in the running container.
4. Start the full-run pipeline with run-pipeline WGS full.
5. Monitor the output from Bpipe (see Bpipe^21^ documentation for more information).
6. When the pipeline exits, you may exit the container with the exit command. All outputs will be located in the working_dir directory on the host machine.

This procedure runs the full end-to-end WGS analysis; you can also run incremental analyses using the run-pipeline command. Enter run-pipeline -h for information about other pre-configured options.

### Procedure: Mouse WES

The procedure for running a Whole Exome analysis on mouse reads is virtually identical to *Protocol: Human WES*, with the following exception:

In step 6, start the full-run pipeline with run-pipeline WES mouse full.

### Procedure: Mouse WGS

The procedure for running a Whole Genome analysis on mouse reads is virtually identical to that given above for human reads, with the following exception:

In step 4, start the full-run pipeline with run-pipeline WGS mouse full.

### Annotations

The oncotator and snpeff directories contain annotation output from Oncotator^10^ and SnpEff^9^, respectively. By default, the consensus UNION is fed into the annotation stage. Annotation can be run on any VCF of interest using the appropriate Bpipe entry point or the run-pipeline helper command.

### QC Information

QC data is collected at several points during the pipeline run. For read QC, see the reads_qc directory. QC on the processed (BAM) alignments is in the bam_qc directory. The variant callers each produce different QC reports, and this data is available in the respective caller’s output subdirectory.

### Storage

The pipeline will place its outputs in the “working” directory (working_dir from above). Within the running container this directory is at the path “/Results”, but it may be located in any convenient location on the host system. Pipeline outputs will be placed in directories labeled to indicate their contents (see Documentation in supplemental materials for directory layout).

To save storage space after the run, it is possible to remove the mapped_reads directory (containing intermediate SAM and BAM files), and the tmp and .bpipe directories. To keep a log of the run, use Bpipe to retrieve the log data with the bpipe log command prior to deleting .bpipe.

### Custom Parameter Settings

The tools used by the pre-configured pipeline analysis procedures have many user-configurable parameters. Most of these have been exposed in configuration files that can be customized on a per-run basis if necessary. As an example, this procedure describes how to retrieve the configuration files and customize them to use a different naming pattern for paired-read *fastq* files.

Assume that the input *fastq* files consist of paired-read files named similarly to “p001_L001_1.fastq.gz” and “p001_L001_2.fastq.gz” where the “_1” and “_2” identify the “left” and “right” siblings.

1. Set up the directory structure as described above for the desired type of analysis.
2. Launch the pipeline container with ./pipeline-start.sh.
3. Run the command get-pipeline-config in the running container.
  This will create a directory configs in the working directory (working_dir/configs on the host machine, and /Results/configs on the running container) if the directory doesn’t already exist.
  The default versions of the files “WES_config.groovy” and “WGS_config.groovy” will be copied into the configs directory (overwriting any version already present there).

4. Shut down the container with the exit command.
5. On the host system, edit the file “WES_config.groovy” and change the line:

- PAIR_PATTERN = “%_R*.fastq.gz”
- To the following:
- PAIR_PATTERN = “%_*.fastq.gz”

5. Save the file.
7. Make the same change to the file “WGS_config.groovy”.
8. Run the pipeline WES or WGS analysis procedure as usual, leaving the config directory with the modified “WES_config.groovy” and “WGS_config.groovy” files in place in the “working” directory on the host. When the analysis is started, the pipeline software will automatically import and use the custom configurations.
  Note that the *container image* will not be modified by this; the next time the container is started, it will still use the default configuration, though custom configurations may be applied as described here at any time. To make a permanent change, it is possible to *commit* the container image after the customized settings files were imported; see Docker documentation for instructions on how to do this.

### Custom Analysis Procedure

To change the pipeline stages performed for one of the pre-configured analyses, edit the Bpipe specification files to specify the new sequence. As an example, this procedure describes how to retrieve the specification files and customize the WES full pre-configured sequence to run only the MuTect2 variant caller.

1. Set up the directory structure as described above for the desired type of analysis.
2. Launch the pipeline container with ./pipeline-start.sh.
3. Run the command get-pipeline-config -s in the running container.

- This will create a directory configs in the working directory (working_dir/configs on the host machine, and /Results/configs on the running container) if the directory doesn’t already exist.
- The default versions of the configuration files will be placed in configs.
- Additionally, a directory configs/pipeline will be created containing the Bpipe sequence scripts (specified in the Groovy programming language), including the file “WES_pipeline.groovy” that will be edited in step 5 below.

4. Shut down the container with the exit command.
5. On the host system, edit the file “WES_pipeline.groovy” and change lines 33–38 from:

- [// Call variants strelka, chr(1.0.22,‘X’,‘Y’) * [mutect2] + chr_vcf_combine] + consensus + annotate
- To the following:
- [// Call variants chr(1..22,‘X’,‘Y’) * [mutect2] + chr_vcf_combine] + annotate

6. Save the file.
7. Run the pipeline WES or WGS analysis procedure as usual, leaving the config directory, config/pipeline sub-directory, and all modified files in place. When the analysis is started, the pipeline software will automatically import and use the custom configurations.
  Note that the *container image* will not be modified by this; the next time the container is started, it will still use the default configuration, though custom configurations may be applied as described here at any time. To make a permanent change, it is possible to *commit* the container image after the customized settings files were imported; see Docker documentation for instructions on how to do this.

### Data availability statement

The information for the pipeline and the datasets used for testing and analysis during the current study is available at: http://bioinformatics.astate.edu/dna-pipeline/.

## Results

**Figure 2:**
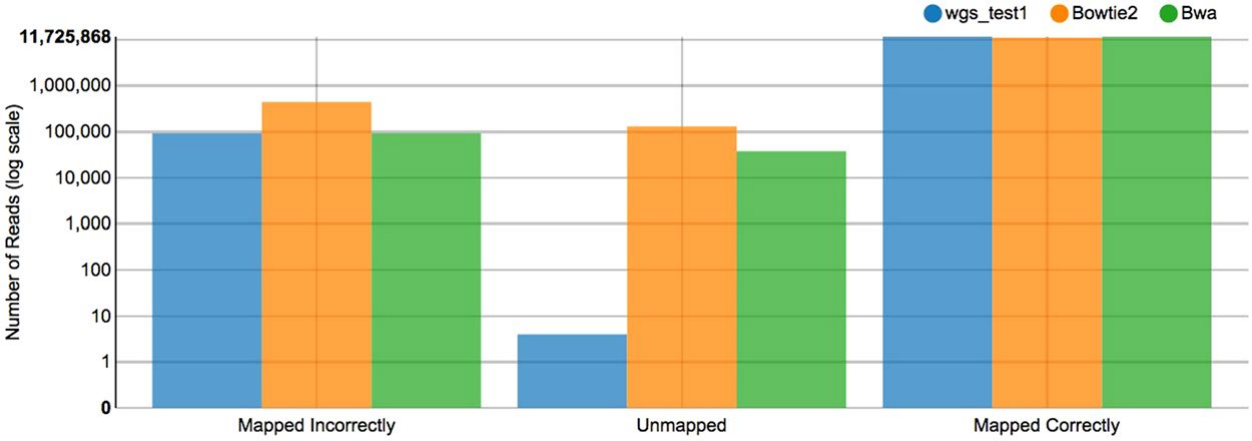
Alignment comparison of A-State Pipeline (blue bars) versus Bowtie2 (orange) and a reference BWA aligner (green) shows similar performance for all three aligners, demonstrating that the pipeline is receiving and aligning the input reads as expected.

Example datasets were used to test the pipeline’s ability to perform its basic functionality, including alignment and somatic variant calling. Alignment was tested using 100 bp read-length paired-end data from the Genome Comparison and Analytic Testing toolkit (GCAT)^22^. Figure 2 shows a comparison of alignment results versus Bowtie2 and BWA aligners as scored by GCAT. Variant calling was tested by comparing the pipeline’s results to results from a publicly-available analysis of human tumor/normal pair HCC1187C/BL from Illumina Basespace (https://basespace.illumina.com/analyses/29429223). Table 2 shows counts for the number of variants matched, unique to the DNAp pipeline’s calling stages, and unique to the reference caller in Whole Exome analysis mode. To simulate a whole-exome capture using the whole-genome dataset, we analyzed only the regions defined in the “SeqCap EZ Exome v3” Human Exome kit by Roche. In the “consensus:union” variant call set, 85% of the reference calls were present in the pipeline output. Variants from dataset ERP00244 (patients 09–129 and 10–497) as reported in a supplemental table by Han *et al*.^23^ were compared to our pipeline results, with 90% of the variants called identically to Han *et al*. Table 3 shows counts for the number of variants matched, unique to the DNAp pipeline’s calling stages, and unique to the reference caller in Whole Genome analysis mode. In this mode 87% of the reference variants were matched in the “consensus:union” output call set. Structural variants called using the dataset “dbGap phs000488.v1.p1” (patient LUAD-2GUGK) in the whole-genome pipeline were compared to Imielinski *et al*.^24^, 26% of the reported structural variants were identically called by our pipeline.

**Table 2:** WES Pipeline test using cell line HCC1187C/BL.

Caller	Matched in Ref	Caller Only	Ref Only
MuTect2	258	1773	47
Strelka	242	24	63
Consensus:Intersection	238	8	67
Consensus:Union	259	1783	46

**Table 3:** WGS Pipeline test using cell line HCC1187C/BL.

Caller	Matched in Ref	Caller Only	Ref Only
MuTect2	13558	116464	2299
Strelka	13180	1821	2677
Consensus:Intersection	12557	273	3300
Consensus:Union	13778	117544	2079

*Mus musculus* (mouse) whole-exome sequencing mode was tested on a selection of variants from the dataset ERP006906 as reported by Nassar *et al*.^25^. The pipeline called 89.8% of selected variants itentically to the results reported by Nassar *et al*.

Output reproducibility on heterogeneous hardware platforms was tested by analyzing the Whole Genome Sequencing data for a patient from the open-access Texas Cancer Research Biobank^26^. Patient *TCRBOA6* was a white female age 61–70 diagnosed with 8246/3: neuroendocrine carcinoma, NOS^26^. The pipeline image was configured and then distributed to two different computing platforms: Site 1: Dell R820 (48 CPU cores, 396 GB RAM, Ubuntu 16.04LTS) on the Arkansas State University campus, and Site 2: Google Compute Engine virtual machine (24 vCPU, 128 GB vRAM, Debian 9). MuTect2 somatic variant calls were 91.0% identical between sites, and 98.8% identical for variants that passed all quality filters. Strelka somatic variant calls were 99.4% identical between sites, and 98.5% identical for variants that passed all quality filters. Lumpy structural variant calls were 99.7% identical between sites. Table 4 shows counts for the number of variants matched, variants unique to Site 1, and variants unique to Site 2.

**Table 4:** WGS Pipeline variant calling test for TCRBOA6 at two heterogeneous sites.

Caller	Filter	Both Sites Matched	%	Site 1 Only	%	Site 2 Only	%
MuTect2	all	184346	91.0	9204	4.5	8973	4.4
	PASS	3700	98.8	23	0.6	23	0.6
Strelka	all	26731	99.4	87	0.3	85	0.3
	PASS	1409	98.5	12	0.8	10	0.7
Lumpy	*	7228	99.7	12	0.2	9	0.1

Site 1 was a Dell R820 server on-site, Site 2 was a Google Compute Engine virtual machine. *Lumpy outputs do not include filter information.

## Discussion

We developed the DNAp pipeline for WGS/WES analysis and tested with human and mouse sequencing data, with a focus on enabling researchers working on different workstations within a research site or even across multiple research sites to collaborate without introducing inconsistencies due to differences in software platforms, versions, or parameter settings. The pipeline is intended to be configured at the outset of the project and “frozen” as a Docker image that is then distributed to each workstation or site. This ensures that every researcher is performing the same analysis with the same software versions and parameters across the entire project. We provide a table listing the non-default parameter settings for each tool in our testing configuration in our supplementary materials. Each research team would need to carefully consider parameter settings appropriate to their research goals when performing the initial configuration.

Likewise, the software tools included in the pipeline were selected to fulfill the needs of our research collaborators, and might not be suitable for the research goals of other projects. However, the tools and parameters can be customized to fit the needs of other researchers, and our pipeline may be used as a template and a learning tool for developing custom container-based pipelines. For example, a different alignment tool (such as Bowtie2) might be substituted for BWA-MEM, or an additional annotation tool such as ANNOVAR^27^ might be added to provide functional predictions. These modifications can be made by an experienced pipeline developer, then the modified pipeline can be executed by relatively inexperienced end-users in the lab. Our pipeline does not offer the same ease of configuration as Galaxy, Seven-Bridges, or ExScalibur, but it serves a different need, allowing groups of collaborators to operate on multiple platforms with an identical containerized toolchain.

We performed throughput tests on human and mouse data and compared with published results. In our alignment test, our performance nearly matched the baseline for the BWA aligner, which was anticipates, as a version of BWA (BWA-MEM) is the tool used in our pipeline. Some differences with the Bowtie2 tool are to be expected due to the difference in alignment algorithms. In our small-variant calling tests, we matched a majority of the published variants, but there were also some differences. It has been shown by Alioto *et al*.^28^ and Krøigård *et al*.^29^ that large differences between variant callers are not uncommon. We believe the differences in our testing results are likely due to a combination of differences in the preprocessing steps between the FASTQ stage and variant-calling, and the different variant callers used. Since our goal was self-consistent performance across multiple sites, we did not attempt to tune parameters to more closely match the published results. Our results did not match well with the published results in the structural variant calling task, where we compared our results on patient LUAD-2GUGK to Imielinski *et al*.^24^. We were able to exactly match 26% (12:46) of the variant calls from the reference paper, although we also called 5 additional positions as a different type of structural variant than reported. We believe the low concordance is likely due to the difference in variant callers: Our pipeline utilized Lumpy for this particular test, while Imielinski *et al*. utilized a custom analysis program they named “Firehose”^24^. Additionally, the Imielinski study originally reported and removed an artifact involving oxidation of guanine bases during sequencing; we did not take any steps to remove this artifact during our test.

We performed comparison tests on two different hardware platforms to determine the pipeline’s ability to produce consistent results on heterogeneous host platforms. We consider this to be the most representative test of the DNAp pipeline, based on our goal of a platform-agnostic tool with consistent outputs. We performed identical WGS analysis at two different sites using FASTQ inputs from patient *TCRBOA6*, from the open-access Texas Cancer Research Biobank^26^. We chose a Dell R820 with 48 CPU cores and 396 GB RAM running Ubuntu 16.04LTS to represent the hardware a lab might have “on-site” (Site 1). For our second site (Site 2), we used a Google Compute Engine virtual machine with 24 virtual CPU cores and 128 GB of RAM allocated. The pipeline was used to produce variant calls from MuTect2, Strelka, and structural variant calls from Lumpy. Our results show >90% agreement between the sites on all outputs (and >98% agreement on all calls that received “PASS” filter messages in the VCF output). See Table 4 for a listing of the number of variants that were called identically versus those that were unique to each site.

The small difference in outputs was traced back to slightly different alignments produced by the BWA-MEM alignment tool. We found similar reports online (https://www.biostars.org/p/90390/ and https://www.biostars.org/p/132277/) dating back to 2014, indicating that the issue is related to random number seeding in BWA and could be dependent on the number of parallel threads allocated to each process. Even though containerization can remove software platform variability from the list of reproducibility challenges, issues like randomization and hardware differences are beyond the ability of the container to standardize and may introduce small differences in the result. If identical results are required, we recommend investigating different alignment tools, or reducing the number of threads so that all sites use the same number of threads, even though this means limiting performance to the level of the least-capable hardware.

We have prepared a Docker image bundle containing the pipeline and made it available to the public. This pipeline is for those users, who have no prior knowledge of pipelines but want to use the pipeline for biomedical discovery, and also for those users, who have experience and prior knowledge and have been working on pipeline development and pipeline comparisons. The development of this open source pipeline is to provide “convenience” to users of the research community, with or without prior knowledge of DNA-seq pipelines, from either individual research groups or large research collaborations.

## Electronic supplementary material

Supplementary information
