# Supplementary material for: DNAp: A Pipeline for DNA-seq Data Analysis
Source: Sci Rep. 2018 May 1;8:6793. doi: 10.1038/s41598-018-25022-6 (PMC5931599; doi:10.1038/s41598-018-25022-6)
Supplement: Supplementary file 1 — Supplementary information [file 41598_2018_25022_MOESM1_ESM.docx]

DNAp: A Pipeline for DNA-seq Data Analysis

Jason L Causey^1^, Cody Ashby^2^, Karl Walker^3^, Zhiping Paul Wang^4^, Mary Yang^5^, Yuanfang Guan^6^, Jason Moore^4^, Xiuzhen Huang^1*^

# SUPPLEMENTARY MATERIAL

## Container Image and Reference Data

The following directory structure must be present on the host machine:

[+] working_dir/
[+] configs/
[f] WES_config.groovy (optional)
[f] WGS_config.groovy (optional)
[ ] [...] (additional pipeline control scripts)
[+] fastq/
[+] normal/
[ ] [...] (normal fastq.gz files)
[+] tumor/
[ ] [...] (tumor fastq.gz files)
[+] tmp/
[f] bpipe.config (optional)
[+] reference_dir/
[+] hg19/
[ ] [...] (hg19 reference files)
[+] mouse_GRCm38/
[ ] [...] (GRCm38 reference files)

A reference bundle is also available from the software download site; it will expand to the correct directory configuration.

At container launch, the "working" (working_dir) and "reference" (reference_dir) directories should be mounted into the container at specific filesystem paths (working_dir ==> /Results and reference_dir ==> /ReferenceData). The pipeline-start.sh script automates this step.

The *working directory* should contain a configs directory for custom configuration files, a directory containing the input data for the pipeline, and a tmp directory that will be used by several tools for transient storage.

For tumor/normal runs, the pipeline must identify the difference between "tumor" inputs and "normal" inputs. By default, simply having the word "tumor" anywhere in the input file's path marks it as a tumor sample, and having the word "normal" in the path marks a file as a normal sample. The easiest way to organize files to satisfy this requirement is to have the tumor and normal sub-directories of the data directory as shown.

The pipeline expects paired-read data, with a *fastq* file for each "end" of the paired read. The naming convention that the pipeline expects by default is that the two paired *fastq* files will contain "_R1" and "_R2", respectively, in their filenames. So the following pair of files would be recognized: "SAMPLE001_L001_R1.fastq.gz" and "SAMPLE001_L001_R2.fastq.gz" Note that the two names are identical except for the "pair tags" _R1 and _R2. If file pairs are identified differently, the configuration files "WES_config.groovy" or "WGS_config.groovy" may be customized to indicate the correct pattern. Default versions of these files may be retrieved in the running container with the get-pipeline-config command.

**VCF Consensus and Merging**

The pipeline is configured to produce consensus VCF outputs in addition to the outputs normally produced by each of the included variant callers. The following consensus outputs are produced: Union of all small variant caller outputs (“consensus:union” available in WES and WGS modes), Intersection of all small variant caller outputs (“consensus:intersection” available in WES and WGS modes), Union of all structural variant callers (“consensus:sv_union”, WGS mode only), Intersection of all structural variant callers (“consensus:sv_intersection”), Union of the “consensus:union” and “consensus:sv_union” call sets (“consensus:union:union”, WGS only), and Union of the “consensus:intersection” and “consensus:sv_union” call set (“consensus:intersection:union”, WGS only). Since some structural variants cannot be represented effectively in VCF format, we recommend also examining the raw outputs of each of the structural variant callers, which are retained in the “structural_variants” output directory.

The process for producing each of the consensus outputs is detailed below.

**consensus:union**

The small variant union is produced by the method:

- - - - 1. Copy all VCF outputs from the small variant callers to a single directory.
        2. Run Jacquard to merge all VCFs in the directory and produce an intermediate output VCF.
        3. Correct the format of Jacquard’s output VCF to match the input requirements of Picard SortVcf by running custom Python scripts. (These scripts are available in the ‘/usr/local/lib’ directory of the container image.)
        4. Run Picard Tools SortVcf on the intermediate VCF file to produce a sorted VCF file. This file is the output of the “consensus:union” method.

**consensus:intersection**

The small variant intersection is produced by the method:

- 1. Using the “consensus:union” VCF as input, run GATK SelectVariants to select the intersection. The output of this step is the output of the “consensus:intersection” method.

**consensus:sv_union**

The structural variant union is produced by the method:

- - 1. Using GATK CombineVariants, create the union of each VCF representation of each structural variant caller. The output of this step is the output of the “consensus:sv_union” method.

**consensus:sv_intersection**

The structural variant intersection is produced by the method:

- - - 1. Using the output of “consensus:sv_union” as input, use GATK SelectVariants to select the intersection. The output of this step is the output of the “consensus:sv_intersection” method.

**consensus:union-union**

The union of the “consensus:union” and “consensus:sv_union” sets is produced by the method:

- - - - 1. Copy the VCF output from “consensus:union” and “consensus:sv_union” into a temporary directory.
        2. Correct the format of Jacquard’s output VCF to match VCF format standards by running a custom Python script. (This script is available in the ‘/usr/local/lib’ directory of the container image.) The output of this stage is the output of the “consensus:union-union” method.

**consensus:intersection-union**

The union of the “consensus:intersection” and “consensus:sv_union” sets is produced by the method:

Copy the VCF output from “consensus:intersection” and “consensus:sv_union” into a temporary directory.

Correct the format of Jacquard’s output VCF to match VCF format standards by running a custom Python script. (This script is available in the ‘/usr/local/lib’ directory of the container image.) The output of this stage is the output of the “consensus:intersection-union” method.

## SUPPLEMENTAL FILES

Container_Reference_Requirements_and_VCF_Consensus_Method_Descriptions.docx

ERP00244_variant_call_validation.xlsx

HCC1187_snp-indel_caller_table.xlsx

LUAD-2GUGK_sv_validation.xlsx

mouse_variant_call_validation.xlsx

parameters_used_in_full_run.xlsx

pipeline_flowchart.pdf

Tables.xlsx

Two-Site_WGS_vcf-compare.zip

**Note**: All supplemental information for this paper "DNAp: A Pipeline for DNA-seq Data Analysis" is available by downloading the ZIP archive at: <http://bioinformatics.astate.edu/dna-pipeline/> .
